# Supplementary material for: Identification and characterisation of hypomethylated DNA loci controlling quantitative resistance in Arabidopsis
Source: eLife. 2019 Jan 4;8:e40655. doi: 10.7554/eLife.40655 (PMC6342528; doi:10.7554/eLife.40655)
Supplement: Transparent reporting form [file elife-40655-transrepform.docx]

***eLife’s* transparent reporting form**

We encourage authors to provide detailed information *within their submission* to facilitate the interpretation and replication of experiments. Authors can upload supporting documentation to indicate the use of appropriate reporting guidelines for health-related research (see [EQUATOR Network](http://www.equator-network.org/%20)), life science research (see the [BioSharing Information Resource](https://biosharing.org/)), or the [ARRIVE guidelines](http://www.plosbiology.org/article/info:doi/10.1371/journal.pbio.1000412) for reporting work involving animal research. Where applicable, authors should refer to any relevant reporting standards documents in this form.

If you have any questions, please consult our Journal Policies and/or contact us: [editorial@elifesciences.org](mailto:editorial@elifesciences.org).

**Sample-size estimation**

- You should state whether an appropriate sample size was computed when the study was being designed
- You should state the statistical method of sample size computation and any required assumptions
- If no explicit power analysis was used, you should describe how you decided what sample (replicate) size (number) to use

Please outline where this information can be found within the submission (e.g., sections or figure legends), or explain why this information doesn’t apply to your submission:

The statistical power of the epiQTL analysis was primarily determined by the size and composition of the epiRIL population, which is based on previous studies about variation in growth phenotypes and DNA methylation. Sample size in subsequent experiments were determined on the basis of previously optimized bio-assays, as well as practical considerations with regards to growth space and seeds availability.

**Replicates**

- You should report how often each experiment was performed
- You should include a definition of biological versus technical replication
- The data obtained should be provided and sufficient information should be provided to indicate the number of independent biological and/or technical replicates
- If you encountered any outliers, you should describe how these were handled
- Criteria for exclusion/inclusion of data should be clearly stated
- High-throughput sequence data should be uploaded before submission, with a private link for reviewers provided (these are available from both GEO and ArrayExpress)

Please outline where this information can be found within the submission (e.g., sections or figure legends), or explain why this information doesn’t apply to your submission:

Information about population sample size and definitions of biological versus technical replication are detailed for each experiment in the Methods section. No data were excluded from the reported analyses and where missing values occur in the source data files, an explanatory note is included. High-throughput sequencing data have been uploaded onto the European Nucleotide Archive (ENA) with reference to the corresponding accession number in the Methods section.

**Statistical reporting**

- Statistical analysis methods should be described and justified
- Raw data should be presented in figures whenever informative to do so (typically when N per group is less than 10)
- For each experiment, you should identify the statistical tests used, exact values of N, definitions of center, methods of multiple test correction, and dispersion and precision measures (e.g., mean, median, SD, SEM, confidence intervals; and, for the major substantive results, a measure of effect size (e.g., Pearson's r, Cohen's d)
- Report exact p-values wherever possible alongside the summary statistics and 95% confidence intervals. These should be reported for all key questions and not only when the p-value is less than 0.05.

Please outline where this information can be found within the submission (e.g., sections or figure legends), or explain why this information doesn’t apply to your submission:

For each experiment and analysis, information about statistical tests, statistical thresholds and false discovery rate corrections are detailed in the Methods section, as well as in each relevant figure legend. Legends of figures presenting results of bio-assays also make reference to the nature of the presented values and error bars.

(For large datasets, or papers with a very large number of statistical tests, you may upload a single table file with tests, Ns, etc., with reference to sections in the manuscript.)

**Group allocation**

- Indicate how samples were allocated into experimental groups (in the case of clinical studies, please specify allocation to treatment method); if randomization was used, please also state if restricted randomization was applied
- Indicate if masking was used during group allocation, data collection and/or data analysis

Please outline where this information can be found within the submission (e.g., sections or figure legends), or explain why this information doesn’t apply to your submission:

Units of biological replication for gene expression and DNA methylation analyses are detailed in the Methods section. Similarly, units of biological replication for the different patho-assays are detailed in the Methods section. Typically, units of biological replication were grouped by tray, containing multiple pots with different plants of different (epi)genotype. Each tray contained at least one control sample (i.e. un-treated and/or Wild-type). The position of plant pots within trays were randomized before treatment, and trays were regulatory rotated within the growth space to prevent positional effects. For salt tolerance assays, quantification of growth stage at 6 dpi was determined on agar plates, each containing different (epi)genotype and salt combinations.

**Additional data files (“source data”)**

- We encourage you to upload relevant additional data files, such as numerical data that are represented as a graph in a figure, or as a summary table
- Where provided, these should be in the most useful format, and they can be uploaded as “Source data” files linked to a main figure or table
- Include model definition files including the full list of parameters used
- Include code used for data analysis (e.g., R, MatLab)
- Avoid stating that data files are “available upon request”

Please indicate the figures or tables for which source data files have been provided:

Source data have been added for: Figure 1; Figure 1-figure supplement 2; Figure 1-figure supplement 3; Figure 1-figure supplement 4; Figure 1-figure supplement 5; Figure 2a, 2b and 2c; Figure 3c. Additional information related to different Figures, but not part of source data, was added in the “Supplementary Datasets” file, and referenced in the main text of the manuscript.
